# Molecular Creep Induced Fatigue Rupture of Fibrin Clots

**DOI:** 10.1002/advs.202505109

**Published:** 2025-08-07

**Authors:** Dani Liu, Zichang Jia, Binchao Liu, Jizhe Hou, Rui Bao, Mingkun Wang

**Affiliations:** ^1^ Institute of Solid Mechanics School of Aeronautic Science and Engineering Beihang University Beijing 100191 China; ^2^ Department of Interventional Radiology, Vascular Surgery Peking University Third Hospital Beijing 100191 China; ^3^ Hangzhou International Innovation Institute Beihang University Hangzhou 310015 China

**Keywords:** embolization, fibrin, multiscale mechanics, soft mechanics, α‐to‐β transition

## Abstract

Fibrin networks, the structural framework of thrombi, undergo fatigue failure under pulsatile blood flow, posing risks of embolic events like stroke, pulmonary embolism. While fibrin's fracture behavior under monotonic loading has been intensively explored, its fatigue mechanism that governs thrombus embolization is understudied. Multiscale experiments, modeling of condensed fibrin networks, reveal a paradoxically low fatigue threshold despite high fracture energy. Mechanistically, cyclic loading induces irreversible α‐to‐β transitions in fibrin's molecular chains, causing molecular creep that dissipates strain energy, propagates cracks via rupture of stress‐localized fibers. A multiscale continuum model quantitatively links this nanoscale mechanics to macroscale fatigue, providing an alternative mechanism of thrombus rupture as a molecular disorder driven by cumulative conformational damage. These findings reexamine widely adopted fibrous hydrogel design paradigms while bridging molecular structure characteristics to clinical thrombosis–a critical step toward predictive rupture risk assessment, targeted therapeutic strategies.

## Graphical abstract

Fibrin networks in thrombi paradoxically exhibit low fatigue resistance despite high fracture energy, as cyclic loading triggers irreversible α‐to‐β transitions in fibrin molecules, causing molecular creep, crack propagation. Multiscale experiments, modeling reveal how nanoscale conformational damage accumulates, linking molecular disorder to macroscale thrombus rupture.

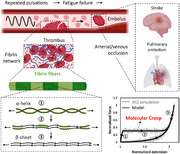

## 1 Introduction

**Figure 1:**
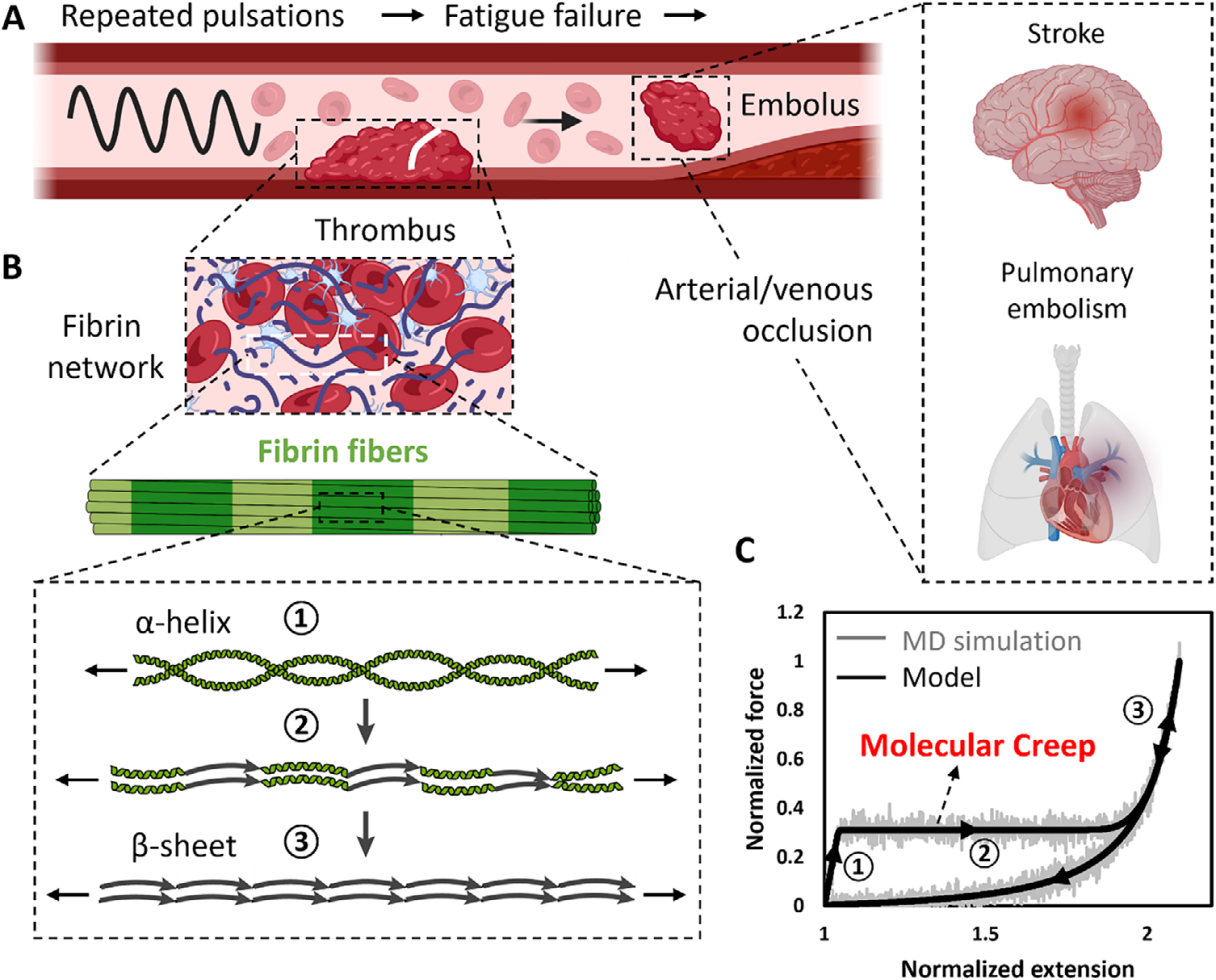
Fatigue failure of fibrin clots, fibrin's multiscale mechanics. A) Fibrin clots experience cyclic loading, potentially leading to fatigue failure, and subsequent embolus formation. Arterial emboli may cause occlusions that lead to strokes, whereas venous emboli may result in pulmonary embolism through venous occlusion. B) Hierarchical architecture of fibrin networks, its conformational transition from α‐helix to β‐sheet. C) Molecular mechanics of α‐toβ transition: 1) initial α‐helix dominated linear elasticity, 2) force‐independent molecular creep during transition, 3) β‐sheet mediated nonlinear elasticity.

Blood clots, composite materials of fibrin networks, and blood cells rely on fibrin's structural hierarchy to maintain mechanical stability under physiological stresses.^[^ ^1^, ^2^ ^]^ Within the vasculature, thrombi (intravascular clots) experience cyclic mechanical loading from pulsatile blood flow (**Figure** **1**A), a process that progressively weakens fibrin networks through fatigue damage. This cumulative degradation ultimately leads to thrombus rupture, embolization – a life‐threatening clinical event where dislodged fragments occlude distal vessels, triggering ischemic strokes, pulmonary embolisms.^[^ ^3^, ^4^ ^]^ Despite these conditions accounting for leading global mortality,^[^ ^5^ ^]^ the mechanisms governing fibrin's fatigue failure under cyclic loading have been incompletely understood.

Fibrin networks exhibit a multiscale architectural hierarchy (Figure 1B):^[^ ^6^ ^]^ the network consists of fibrin fibers assembled from aligned fibrils; the fibrils comprise molecular chains containing mechanically responsive α‐helix domains. These domains undergo force‐induced unfolding into β‐sheet conformations,^[^ ^7^ ^]^ enabling extraordinary fiber extensibility (332% ± 71%)^[^ ^8^ ^]^ that influences network mechanics.^[^ ^9^, ^10^, ^11^ ^]^ Molecular dynamics (MD) simulations reveal triphasic force‐extension behavior during this structural transition (Figure 1C)^[^ ^12^, ^13^ ^]^ initial α‐helix dominated linear elasticity, followed by force‐independent molecular creep during α‐to‐β transition, β‐sheet mediated nonlinear elasticity. While this conformational transition explains fibrin's macroscopic extensibility, its role in fibrin fatigue is unclear. Current studies on fibrin fracture mainly focus on fiber, network‐level mechanisms,^[^ ^14^, ^15^, ^16^ ^]^ but how the nanoscale mechanics of conformational transition affects macroscale fatigue remains an open question.

Here we studied the fracture, fatigue behaviors of dense fibrin networks, and explored their correlations with their nanoscale mechanics. Compared with conventional hydrogels,^[^ ^17^, ^18^ ^]^ our measurements of fibrin gels showed a higher fracture toughness of 184 ± 23 J m^−2^ but a low fatigue threshold only of 5.79 J m^−2^. It has been widely acknowledged that fibrous hydrogels are generally fatigue resistant through synchronizing polymer chains in fibers,^[^ ^19^, ^20^, ^21^, ^22^ ^]^ but our data seem to violate this acknowledgement. Such a low fatigue threshold indicates the failure of intra‐fiber chain synchrony, which involves a nanoscale mechanism that leads to fiber deterioration.

Through multiscale analysis, we find that molecular creep, the characteristic nanoscale mechanics of conformational transition, drives fatigue damage in dense fibrin networks. Our multiscale continuum mechanical model quantitatively links molecular creep to energy dissipation, verifying a critical role of nanomechanics in fibrin fatigue. These findings reexamine the presumed universality of fibrous architectures in fatigue‐resistant biomaterials, emphasizing nanoscale mechanics as a critical–yet overlooked–factor of fatigue tolerance. This work establishes a mechanistic bridge between molecular nanoscale mechanics, clinical embolization risks while providing foundational insights for engineering fatigue‐resistant soft materials in biomedical applications.

## 2 Results

One challenge in studying the fatigue behavior of soft materials, especially biological materials, is experimental variation. To minimize the artificial variations caused by material composition, we used “synthetic fibrin hydrogels” prepared under controlled conditions, instead of using in vivo blood clots. We controlled the clotting conditions, including fibrinogen concentration, fibrinogen/thrombin ratio, clotting temperature, and time. These fibrin gels produced consistent, reproducible data, have been proven to replicate the mechanical properties of blood clots.^[^ ^1^, ^2^ ^]^

### 2.1 Fibrin Gels were Susceptible to Fatigue Damage Despite Fibrous Architecture

To demonstrate the fundamental differences between fracture, fatigue, we performed pure shear tests with two types of loading: monotonic tensile loading (a single stretch), cyclic fatigue loading (repeated loading).

**Figure 2:**
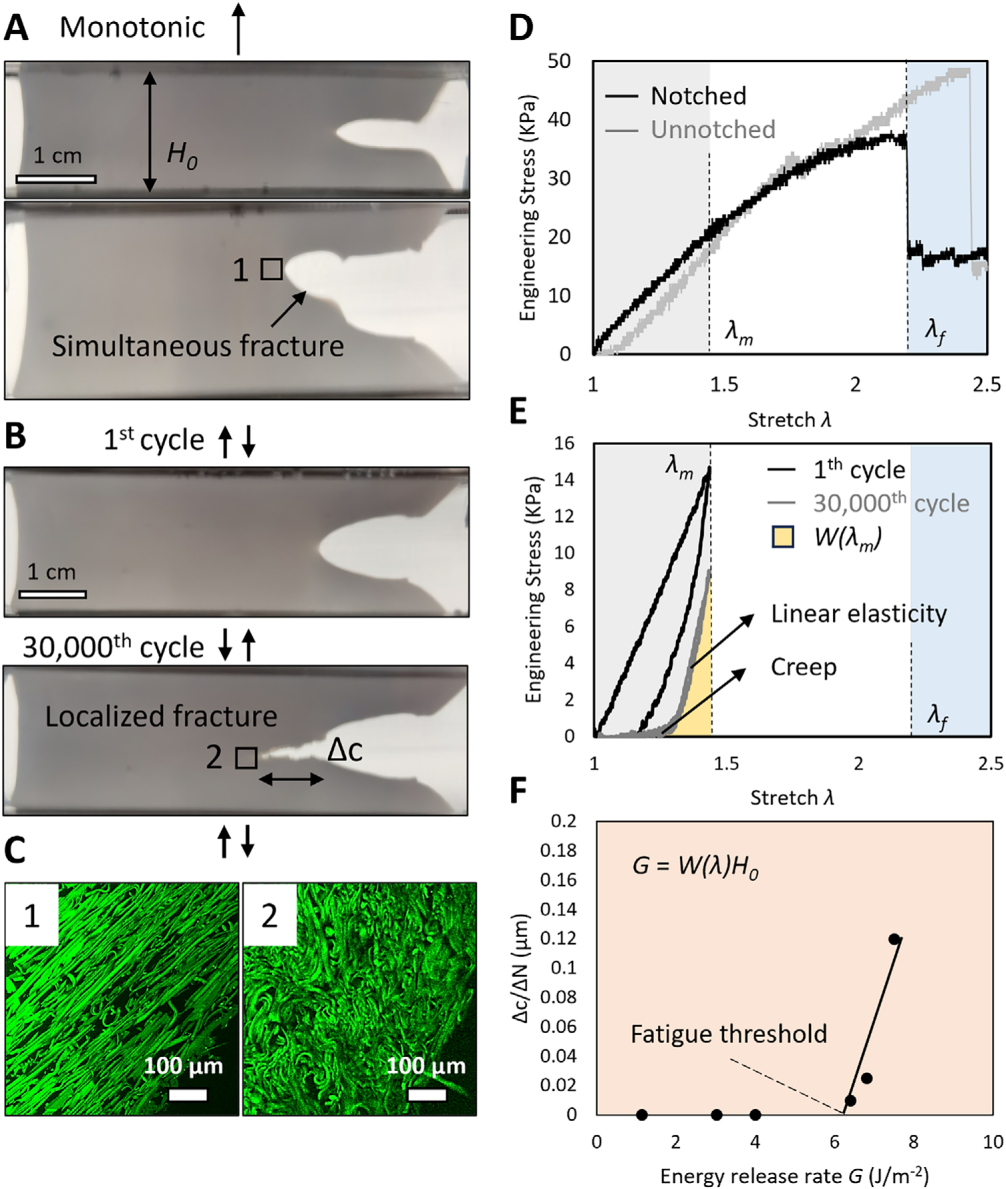
Fibrin gels exhibited a low fatigue threshold. Crack propagation in fibrin hydrogels A) under monotonic loading, B) under cyclic loading. C) Confocal fluorescence images displaying the fiber morphology next to the crack tip. D) Engineering stress‐stretch curves of fibrin hydrogels under monotonic tensile loading. E) Engineering stress‐stretch curves for the first, 30 000th loading‐unloading cycles, the curve of 30 000th cycle showed a bilinear behavior with a creep regime, a linear elastic regime. The yellow shaded area represents strain energy density *W(λ)*. F) Fatigue threshold is the maximum energy release rate *G* at *dc/dN* = 0, where *c* is the crack length, *N* is the number of cycles. *λ_m_* represents the minimal stretch for the crack to propagate, which is also the maximal stretch for *dc*/*dN *= 0.

Under monotonic tensile loads, the fibrin fibers effectively arrested crack propagation. The resulting fracture surface was smooth, wide, indicating that a large region of fibers fractured together (**Figure** **2**A). This resulted in a fracture point at stretch *λ_f_* = 2.17 ± 0.15 for the notched samples, which is close to the ultimate tensile stretch *λ_u_* = 2.48 ± 0.22 measured by uniaxial tensile test (Figure 2D).

Nevertheless, this ‘fiber effect’ waned under cyclic fatigue loads. The fatigue fracture surface was sharp, and the crack advanced merely by fracturing a single layer of fibers at its tip (Figure 2B). As a result, crack propagation even at a stretch of 1.46, which was remarkably lower than *λ_f_* = 2.17 ± 0.15 (Figure 2E).

A closer inspection of fiber morphology provided further insight into these contrasting behaviors (Figure 2C). Under monotonic loading, fibers aligned to bear the load collectively. This represents a typical failure mode of a fibrous structure when aligned fibers fracture simultaneously. In contrast, under fatigue loading, fibers adopted a curled configuration, suggesting that most fibers did not effectively carry the load. This indicated a localized fiber fracture at the crack tip, distinct from the typical failure mode of simultaneous fracture.

These observations pointed out the essential difference between fracture, fatigue in fibrin hydrogels: fibrin hydrogels were much more susceptible to fatigue damage. Unlike monotonic loading where all fibers aligned, collectively bore the load, under fatigue conditions, most fibers did not participate in the loading‐bearing, leading to localized damage, and subsequent crack propagation.

### 2.2 Fibrin Gels Exhibited Low Fatigue Threshold

Next, we continued to quantify the resistance to fracture, fatigue using the pure shear tests (Figure S1, Supporting Information). Fracture toughness is defined as the energy required to propagate a crack per unit area in the material's undeformed state. Fatigue threshold is defined as the minimal fracture energy at which fatigue crack propagation occurs under infinite loading cycles.

For the pure shear tests, specimens were notched, designed with a width significantly greater than the grip separation, which itself was much larger than the sample thickness. This setup aimed to create a simple stress field ahead of the crack, leading to more consistent experimental data. Given that viscoelastic materials are sensitive to strain rate, we performed cyclic loading tests at rates of 0.5, 1, and 2 s^−1^. Monotonic loading tests used the same loading rate to ensure comparability with the fatigue results. All tests were conducted in deionized water to prevent dehydration‐induced embrittlement of hydrogels during fatigue testing.

The measurement of fracture toughness was straightforward, involving the multiplication of strain energy density *W*(*λ*), the original height between grips *H*_0_, using an unnotched sample stretched to *λ_f_*. Our measurement showed that the fracture toughness of 10 wt.% fibrin hydrogels was 184 ± 23 J m^−2^, significantly higher than that of conventional brittle hydrogels.

In terms of fatigue threshold. We recorded crack growth rates during each loading cycle *dc*/*dN* (Figure S2, Supporting Information), calculated the corresponding energy release rate *G* by multiplying the strain energy density *W*(*λ*) by the original height between grips *H*_0_. The area under the last loading cycle's stress‐stretch curve (the yellow area in Figure 2E) represented *W*(*λ*). The fatigue threshold, defined as the maximum energy release rate *G_m_* for which *dc*/*dN* = 0, was measured ≈5.79 J m^−2^ (Figure 2F). This value is not only far below those of load‐bearing biological hydrogels but also modest compared to conventional brittle hydrogels.

A notable ‘shake‐down’ phenomenon was observed in the stress‐stretch curves of samples during cyclic loading (Figure 2E): compared to the first loading‐unloading cycle, the 30 000th cycle showed a bilinear behavior–a creep line (increased stretch without increase in stress), a linear elastic line.

### 2.3 Macroscale Mechanisms

**Figure 3:**
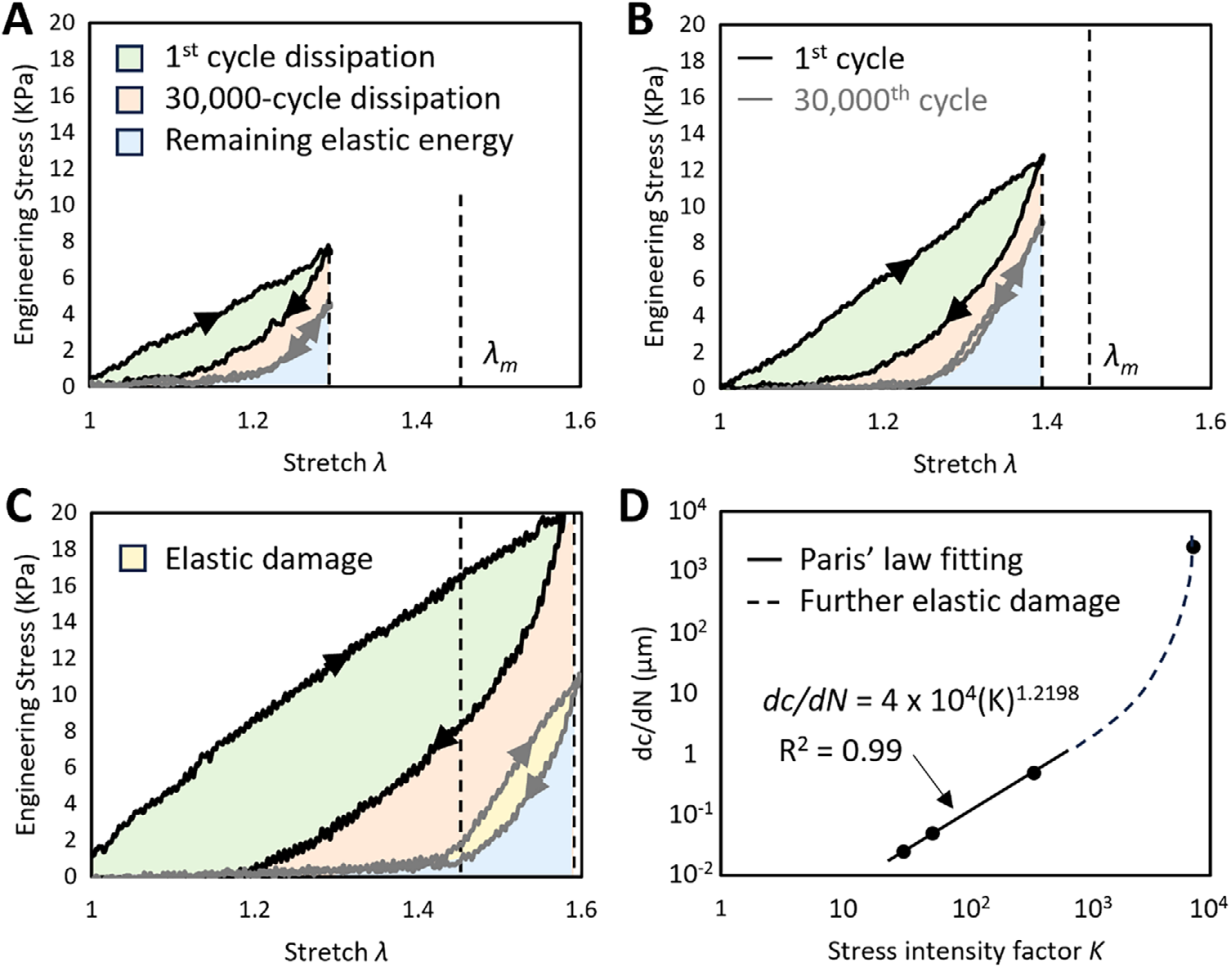
Macroscale damage, fracture mechanics. Engineering stress‐stretch curves of the first, 30 000th loading‐unloading cycles when loaded in cyclic fatigue to A) *λ *= 1.3. B) *λ *= 1.4. C) *λ *= 1.6. D) Crack propagation rate, stress intensity factor, followed Paris’ law, prior to the accumulation of damages.

To explore the fatigue mechanism, we examined the energy dissipation, damage accumulation during the 30 000 cycles of fatigue loading. On the stress‐stretch curves, the total strain energy density can be partitioned into four regimes (**Figure** **3**A): the green area represents the energy dissipated by the first loading cycle, while the red area represents the energy dissipated by the following 30 000 loading cycles; after 30 000 cycles, the remaining blue area is the recoverable elastic energy; further loading to *λ* > *λ_m_* (*λ_m_* represents the minimal stretch for crack to propagate, which is also the maximal stretch for *dc*/*dN *= 0) creates damage to fibrin fibers, eventually leads to crack propagation, this damage is represented as the strain energy density in yellow.

We analyzed the stress‐stretch curves of samples subjected to cyclic loading at representative stretches: *λ* = 1.3 (where the creep line was not yet prominent); *λ* = 1.4, *λ* = 1.44, *λ* = 1.5, (near *λ_m_*);, *λ* = 1.6, (exceeding *λ_m_*). It is obvious that most energy was consumed by cyclic loading, giving a longer, longer creep stretch (Figure 3A–C). Collectively, the energy dissipation played a crucial role in the low *W*(*λ_m_*), fatigue threshold, accounting for 75% to 85% of the total energy (Figure S3A, Supporting Information). Considering the strain rate sensitivity of fibrin hydrogels, we repeated tests at rates of 1, 0.5, and 2 s^−1^. As anticipated, the strain rate significantly influenced the mechanical response under monotonic loading (FigureS3B, Supporting Information), but only marginally affected the dissipation ratios without altering their overall trends. These results were not unexpected, as the energy dissipation, in contrast to the transient mechanical response, represents the steady‐state characteristics.

In addition to a linear creep regime, the stress‐stretch curves of the 30 000th cycle exhibited another linear regime (between *λ* = 1.4, *λ* = 1.5), which represented a linear elasticity (Figures 2E and 3A–C). This linear elasticity was probably because of the branch points. Even when fibers were loosened, the branching networks could still provide a simple linear elasticity. This linear elasticity was also verified in fracture mechanics. The relationship between crack propagation rate *dc/dN*, stress concentration factor *K* follows to Paris’ law (Figure 3D), a principle traditionally used to describe fatigue crack growth in linear elastic materials. These data together suggested a linear elasticity of fibrin networks after the large energy dissipation.

### 2.4 Mesoscale Mechanisms

**Figure 4:**
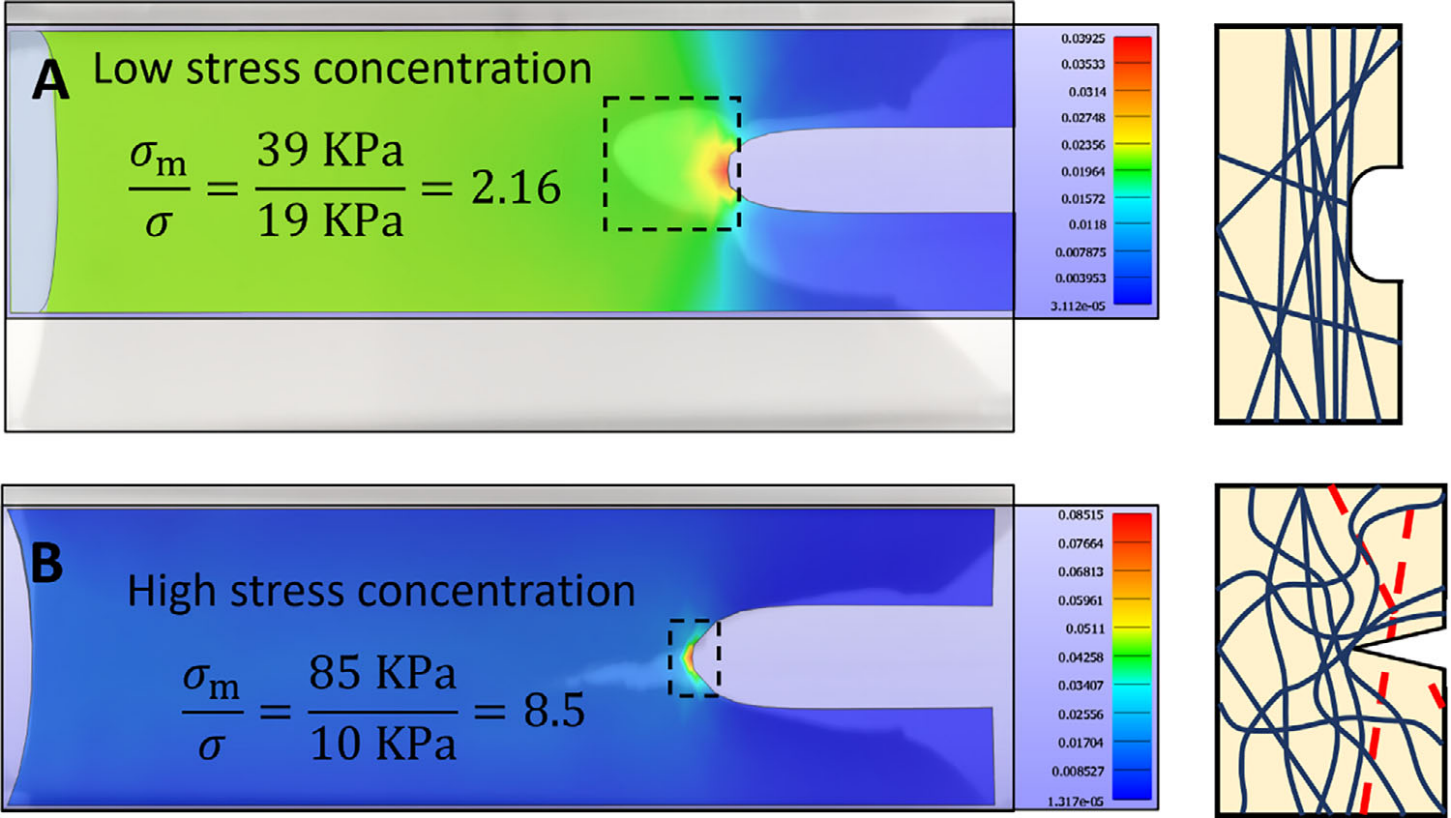
Network‐level stress concentration facilitated crack growth. Finite element analysis was used to simulate pure shear tests, with a stretch to 1.4. A) For the monotonic loading simulation, the material was modeled as a continuous fiber model. *σ_m_* denotes the maximum stress at the crack tip, *σ* represents the nominal engineering stress, *σ_m_*/*σ* indicates the stress concentration factor. B) When simulating cyclic fatigue loading, a homogeneous hyperelastic model was adopted. The simulation results were superimposed onto the experimental photos.

The large energy dissipation indicates an accumulation of fatigue damage in fibrin networks, the residual linear elasticity significantly lowers the fatigue threshold by increasing stress concentration at the crack tip. To demonstrate the influence of the large dissipation on crack propagation, we used Finite Element Analysis (FEA) to calculate the stress concentrations at the crack tip of dissipated, undissipated samples (**Figure** **4**).

The undissipated specimens were modelled with the stress‐stretch curves from the monotonic fracture test (Figure 2D), adopting a transversely isotropic Mooney‐Rivlin model to fit the data. We then applied the fitted material parameters to model a notched sample under uniaxial tension, with a λ = 1.4. The analysis revealed a stress concentration factor σ_m_/σ = 2.16, with a maximum stress at the crack tip σ_m_ = 39 kPa, the nominal engineering stress σ = 19 kPa (Figure 4A).

The dissipated specimens were modelled with a neo‐Hookean model calibrated to the stress‐stretch curves from the 30 000th loading cycle of the fatigue test (Figure 2E). With the fitted material parameters, we simulated the same notched sample under uniaxial tension to λ = 1.4. Compared with undissipated samples, the stress concentration factor of dissipated samples reaches σ_m_/σ = 8.5, over four times higher than that of undissipated samples. Even though the nominal stress was only σ = 10 kPa, the maximum stress at the crack tip σ_m_ = 85 kPa—more than double that in undissipated samples (Figure 4B). Notably, the outline shape of FEA results matched exactly the stressed regions in experimental images, verifying higher stress concentrations at the crack tip in dissipated samples.

### 2.5 Nanoscale Mechanisms

**Figure 5:**
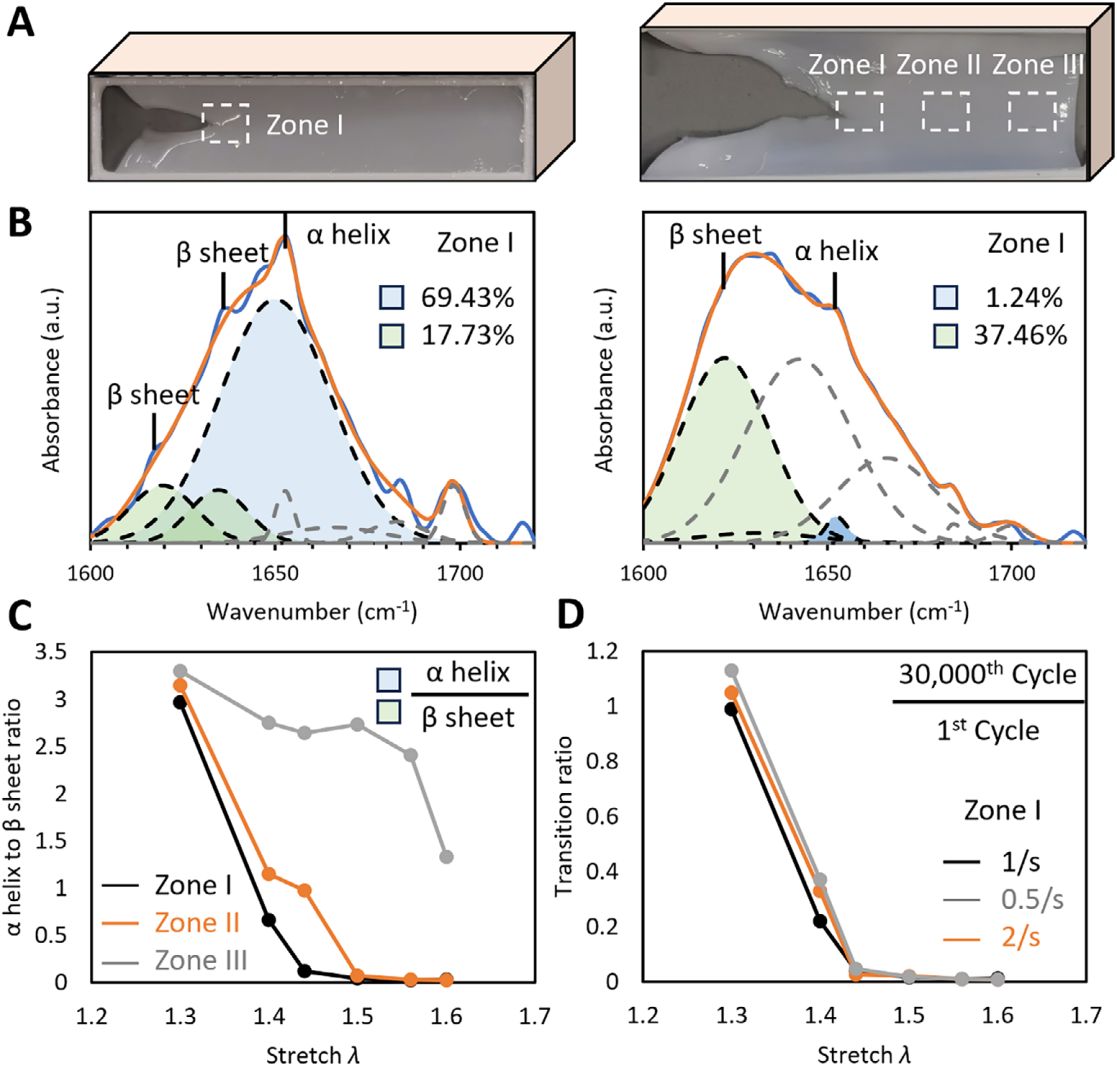
Cyclic loading caused an irreversible α‐to‐β transition. A) Photographic comparison of an intact sample, the same sample after fatigue loading is shown. B) Fourier Transform Infrared (FTIR) spectra were utilized to quantify the α‐helix, β‐sheet content in Zone I before, after exposure to fatigue loading. C) The content ratios of α helices to β sheets across various zones, along with their correlation to the stretch ratio. D) The transition ratios of α helices to β sheets for Zone I, and their relationship with the stretch ratio *λ*.

To further identify the molecular mechanism of fibrin fatigue, we examined the changes in the molecular structure of fibrin due to cyclic loading. To assess the reversibility under cyclic loading, we analyzed the secondary structures in three distinct regions: Zone I at the crack tip, Zone II in the middle region,, Zone III far from the crack (**Figure** **5**A).

Fourier Transform Infrared (FTIR) spectroscopy, a widely accepted method for detecting protein secondary structures,^[^ ^23^ ^]^ including those in fibrin,^[^ ^24^ ^]^ was employed. The FTIR spectra feature multiple bands, with the amide I band being particularly informative due to its association with the C═O stretch of amides. To quantify the content of each secondary structure, the amide I band was deconvoluted into six Gaussian distributions: the band with a peak ≈1655 cm⁻¹ was assigned to α–helices, those with peaks at ≈1620 and 1630 cm⁻¹ were assigned to β‐sheets. By calculating the areas under these Gaussian distributions (Figure 5B), we found that intact fibrin hydrogels predominantly consisted of α helices (≈70%) with a limited number of β sheets (less than 20%). After fatigue loading to *λ_m_*, the content of α helices plummeted to almost undetectable levels, while the content of β sheets surged to ≈40%.

These findings align with previous studies predicting that almost all α helices underwent phase transition by *λ* = 1.6 under uniaxial tensile stretch.^[^ ^7^ ^]^ However, in our study, not all α helices transformed into β sheets; rather, many unfolded into random coils. This transformation into random coils represents a form of molecular damage, contributing to the macroscopic creep behavior.

To quantify the reversibility of the α‐to‐β transition, we examined the ratios of α‐helix to β‐sheet content across different zones. In Zones I, II, the α/β content ratio dropped sharply from *λ* = 1.3 to *λ* = 1.4, and remained minimal thereafter (Figure 5C). Although Zone III, less affected by the crack due to its distance, maintained a higher α‐helix content up to *λ* = 1.6, it also rapidly lost α helices beyond this point. We further calculated the transition ratio—the ratio of α/β content ratios between the 30 000th, 1st cycles (Figure 5D) — which mirrored the trends observed in Zone I at various *λ* values. Importantly, these transition ratios showed insensitivity to strain rates (Figure 5C,D), showing the irreversible nature of the α‐to‐β transition in fibrin.

### 2.6 Molecular creep induced the low fatigue threshold

A variety of factors can contribute to the energy dissipation. To determine the role of irreversible α‐to‐β transition, we developed a numerical model.

#### 2.6.1 At the Molecular Scale–Statistic Mechanical Model

**Figure 6:**
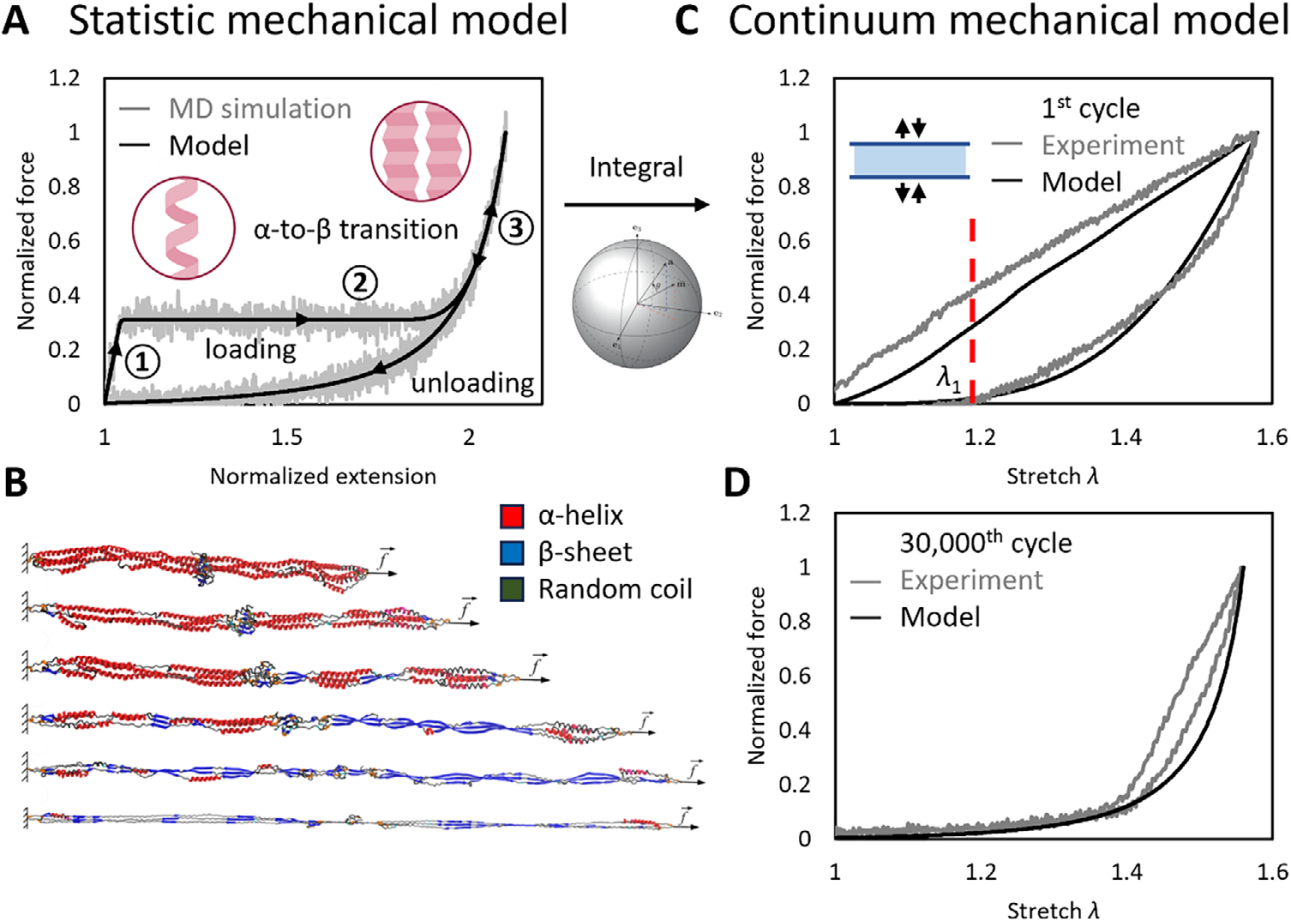
Molecular creep induced the low fatigue threshold. A) A statistical mechanical model describing a single fibrinogen chain undergoing α‐to‐β transition, delineated into three distinct regimes: 1) The α‐helix regime exhibits linear elasticity, adheres to the entropic spring model; 2) The α‐to‐β transition regime displays creep behavior, is characterized by a straight‐line response; 3) The β‐sheet regime shows nonlinear elasticity, conforms to the worm‐like chain model. B) The reported simulation results that illustrate the molecular structural changes from the folded state to the unfolded state, reproduced with permission.^[^ ^26^ ^]^ 2012, ACS Publications. C) The statistical mechanical model was integrated within a full network model to derive a continuum mechanical model of fibrin hydrogels. Comparison of normalized stress‐stretch relations between the developed model, experimental data of the first loading‐unloading cycle. D) Comparison of normalized stress‐stretch relations between the developed model, experimental data of the 30 000th loading‐unloading cycle.

We referred to the published molecular dynamics simulations of a single fibrinogen chain to establish a statistical mechanical α‐to‐β transition framework (**Figure** **6**A).^[^ ^25^, ^26^ ^]^ The atomic structure of these simulations featured double three‐stranded coiled coils. For clarity, we have reprinted snapshots from the published simulation results that illustrate the molecular structural changes from the folded state to the unfolded state (Figure 6B). We chose the data simulated with a speed of 10^7^ nm s^−1^, which was 10 mm s^−1^, consistent with our experiments. Our statistical mechanical model encompasses both loading, unloading curves, with the loading curve divided into three distinct regimes:

The α‐helix regime exhibits linear elasticity, adheres to the entropic spring model:

(1) $$f_{\alpha }\left(\lambda \right)=k_{\alpha }\left(\lambda -1\right)L_{0}$$

where *k*_α_ is the constant stiffness of the entropic spring, *L*_0_ is the starting length;

The α‐to‐β transition regime displays creep behavior; the critical force is dictated by the following expression:

(2) $$\begin{array}{c} f^{*}={\left(\frac{z_{\alpha \beta }}{k_{B}T}-\frac{L_{\alpha m}}{k_{\alpha }\left(L_{\beta m}-L_{\alpha m}\right)}\right)}^{-1}\left(\ln\frac{v_{f}}{A\left(L_{\beta m}-L_{\alpha m}\right)}+\frac{\in_{0}}{k_{B}T}\right) \end{array}$$

where *k_B_* is Boltzmann's constant, *T* is the temperature, *z*_αβ_ is the distance from the α‐state to the transition, *L*_αm_, *L*_βm_ are the maximal extensions in the α‐, β‐states, respectively. *v_f_* is the loading speed, ∈ _0_ is the energy barrier between α‐, β‐states;

During phase transition, the structure is assumed to be in a mixed state of α‐helix, β‐sheet, where the length ratio of α‐helix *R*_α_ in the deformed structure gradually decreases from 1 to 0. The original length of α‐helix *L*_α,0_ co‐varies with *R*_α_,, vice versa for the original length of β‐sheet *L*_β,0_.

The β‐sheet regime shows nonlinear elasticity, conforms to the worm‐like chain model:

(3) $$f_{\beta }\left(\lambda \right)=\left(k_{B}T/4l_{\beta }\right)\left\{{\left[1-\frac{L_{\alpha m}}{L_{\beta m}}\lambda \right]}^{-2}-{\left[1-\frac{L_{\alpha m}}{L_{\beta m}}\right]}^{-2}\right\}$$

where *l*_β_ is the persistence length of β‐state.

When unloading happens, the structure behaves elastically like α‐helix before transition initiates or like β‐sheet after transition is completed. However, if unloading occurs in the middle of transition process, the transition paused, constituent ratio of the structure was frozen. Force is uniform through the structure, equivalent between the two phases:

(4) $$f_{\alpha }\left(\lambda \right)=f_{\beta }\left(\lambda \right)$$

Controlled by the above equation, unloading curves exhibit non‐linearity, history dependency.

#### 2.6.2 At the Fiber Scale

Fibers are an aggregation of fibrils with aligned fibrinogen chains,^[^ ^27^ ^]^ following the above force‐extension relation.

#### 2.6.3 At the Large Scale–Continuum Mechanical Model

The statistical mechanical model was integrated within a full network model to derive a continuum mechanical model of fibrin hydrogels (Figure 6C). The integration was performed using an affine microsphere model with rigid linkers,^[^ ^28^ ^]^ assuming that branching networks between fibers do not break under any conditions.

The detailed statistical mechanical model, integral protocol were included in the supporting information.

#### 2.6.4 Prediction of Macroscopic Irreversible Deformation

The continuum mechanical model successfully captured the hysteresis loop of the first loading‐unloading cycle (Figure 6C), its predicted plastic deformation aligns well with experimental data. The strain energy density enclosed by experimental data exceeded that predicted by the model, largely because the model did not account for other viscoelastic dissipation mechanisms such as water‐out from inner—. and inter—. fibers.

The continuum mechanical model also captured the nonlinear behavior of the 30 000th loading‐unloading cycle (Figure 6D), reflecting the energy dissipation. It precisely predicted a long creep region prior to a steep stiffening segment, although its prediction of the nonlinearity did not fully align with the bilinear behavior of experimental data. The difference in linearity, nonlinearity was mainly due to the neglect of branch points, which store significant elastic energy.^[^ ^29^ ^]^ Our model treated the branch points as rigid, thus underestimating the elastic energy in the bulk material.

Given the lack of calibration with experimental data, this model demonstrated that the irreversible α‐to‐β transition provides a robust explanation for the observed energy dissipation.

## 3 Discussion

For physiological relevance, we used fibrin gels with a fibrinogen concentration of 100 mg mL^−1^ (10 wt.%). While fibrinogen concentration in circulating blood is only 2–4 mg mL^−1^, it locally elevates at the wound site when clotting. Analysis on thrombi showed that fibrin networks account for 70 wt.% dry weight of venous thrombi, 50–60 wt.% dry weight of arterial thrombi.^[^ ^30^, ^31^ ^]^ Given the water content of thrombi is usually between 70 to 90 wt.%, the final concentration of fibrin in thrombi is ≈5–20 wt.%. In addition, as a naturally biocompatible biomaterial, fibrin has been widely used as tissue engineering scaffolds, surgical adhesives, where fibrin weight percentages are normally up to 100 mg mL^−1^.^[^ ^32^ ^]^ Therefore, the dense fibrin gels we used in this study are clinically relevant.

Our measured fracture toughness of fibrin gels is dramatically higher than prior studies,^[^ ^33^, ^34^, ^35^ ^]^ which is typically ≈7.6 ± 0.45 J m^−2^,^[^ ^36^ ^]^ ≈y 24‐fold lower than our measured 184 ± 23 J m^−2^. Despite this large discrepancy, our measurement aligns well with those studies, as fibrin toughness in theory scales with fibrinogen concentration.^[^ ^37^ ^]^ The concentration in our study was 100 mg mL^−1^, 20 to 25‐fold higher than the concentrations used in prior studies (4–5 mg mL^−1^), in agreement with the fold change in toughness.

Compared with the recent study on fibrin fatigue, which measured a threshold of 1.66 J m^−2^,^[^ ^38^ ^]^ the measurement in our study (5.79 J m^−2^) is 3.5‐fold higher. Considering the concentration used in our study again is 20‐fold higher, this increase in fatigue threshold was limited, showing the marginal effect of fibrinogen concentration on fibrin fatigue threshold. Despite the different threshold values, these two studies were in good agreement: the stretches *λ_m_* or strains ε_m_ were in the same range, *λ_m_* in this study is between 1.41 to 1.45, ε_m_ in the prior study is between 0.41 and 0.44. Both studies observe similar failure morphologies with rough fracture surfaces, non‐aligned fibrin fibers at crack tips, indicating a similar mechanism of single fiber fracture across different concentrations.

To interpret the measured fatigue threshold, we estimated the theoretical threshold with an extended Lake‐Thomas model:^[^ ^38^, ^39^ ^]^

(5) $$\Gamma_{0}=\alpha n_{fiber}U_{fiber}L_{fiber}=\alpha n_{fiber}\frac{L_{fiber}^{2}}{r_{0}}N_{p}U_{monomer}$$

where *α* = 3 is the non‐dimensional enhance factor for fibrous polymer networks;^[^ ^40^ ^]^ *n_fiber_* = 2.3 ± 0.1 µm^−3^ is the number of fibers per unit volume of the fibrous network in the swollen state,, quantified from confocal images; *U_fiber_* is the energy required to fracture a single fiber; *L_fiber_* = 1.28 ± 0.27 µm is the average length of the fiber segments between adjacent branching points of the fibrous network in the undeformed state; *r_0_* = 22.5 nm is the length of the regularly repeating unit of protofibril;^[^ ^41^ ^]^ *U_monomoer_* = 2400 kJ mol^−1^ is the energy required to rupture a fiber per monomer;^[^ ^42^ ^]^ *N_p_* is the number of protofibrils per fiber, it decreases as the diameter increases due to activation‐, diffusion‐ limited aggregation effect:^[^ ^43^ ^]^

(6) $$N_{p}=N_{p0}{\left(\frac{d}{d_{0}}\right)}^{\Psi }$$

where *N_p0_* = 400, *d_0_* = 50 nm are the numbers of protofibrils per fiber, fiber diameter of a referring density;^[^ ^44^ ^]^ *Ψ* is the aggregation effect, ranging from 0.4 to 2; *d* = 0.47 ± 0.05 µm is the fiber diameter quantified from confocal images.

Using the above parameters, we estimated *Γ_0_* = 6.34 J m^−2^ when *Ψ* = 0.4, can reach 59.22 J m^−2^ when *Ψ* = 1.4. Considering the dense fibrin networks we used in this study, the aggregation effect *Ψ* > 0.4 would be more relevant, so the theoretical fatigue threshold should be well above our measurement of 5.79 J m^−2^. Since the theory estimated the energy required to rupture a single fiber, this disagreement implies that the fibers at the crack tip became more vulnerable under fatigue loads, highlighting the toughness deterioration of fibrin fibers due to the irreversible molecular structure changes.

Our measurements of fibrin gels showed a high ratio of fracture toughness to fatigue threshold: while the fracture toughness of 10 wt.% fibrin gels is almost one order of magnitude higher than that 10 wt.% Polyacrylamide hydrogel crosslinked with 0.2 wt.% of N,N'‐Methylenebisacrylamide (38.2 ± 3.4 J m^−2^),^[^ ^17^ ^]^ fibrin's fatigue threshold is lower than the Polyacrylamide hydrogels (9.3 J m^−2^).^[^ ^17^ ^]^ This high fracture toughness to fatigue threshold ratio is related to fibrin's function, as it serves as a temporary scaffold for wound healing. Although fibrin requires a high fracture toughness to prevent occasional wound fractures, it does not need a high fatigue threshold like load‐bearing tissues such as muscle collagen networks, whose fatigue threshold is >1000 J m^−2^).^[^ ^45^ ^]^ The ratio of dense fibrin networks is also about twice that of low concentration networks (6–10),^[^ ^38^ ^]^ indicating dense networks may have less crosslinked fibers, despite those fibers are highly aggregated. The aggregated networks tend to have higher toughness, as pulling fibers out of aggregations could dissipate considerable energy, but such dissipation can only work once, contributing little to the long‐term fatigue threshold.

These mechanical insights carry profound clinical implications. Our findings suggest fatigue accumulation predominates over monotonic failure in physiological clot rupture mechanisms. Consider carotid artery hemodynamics: pulsatile flows generate wall shear stresses through velocity fluctuations from 20–40 cm s^−1^ (diastolic) to 80–140 cm s^−1^ (systolic).^[^ ^46^ ^]^ Each cardiac cycle delivers ≈70 µL blood volume at 13.3 kPa mean pressure (*W_0_* = *pV* ≈ 1 J), with 20% (*W_1_* = 0.2 J) reaching carotid arteries. Pressure differentials (*Δp* ≈ 1.3 kPa) across the common carotid yield arterial wall work *W* ≈ 0.02 J (Figure S4, Supporting Information). For typical carotid dimensions (6–8 mm diameter, 10–15 cm length), pulsatile work per unit area becomes 5.3–10.64 J m^−2^ – two orders below fibrin's fracture toughness but marginally exceeding its fatigue threshold. This quantitative alignment explains clinical observations where cyclic loading induces progressive damage rather than immediate rupture, necessitating urgent intervention for carotid clots. Such a mechanistic understanding enables predictive modeling through CFD‐coupled fatigue threshold analysis, potentially contributing to clinical risk assessment.

In addition to the nanoscale mechanics, other mechanisms that cross different scales may also be involved in fibrin fatigue, such as poroelastic dissipation,^[^ ^1^ ^]^ viscoelastic dissipation through interactions between densified fibers.^[^ ^10^ ^]^ The poroelastic effect may be limited, as fibrin gels dehydrate fast with increasing stretch.^[^ ^7^ ^]^ We quantified the changes in thickness, volume of samples under cyclic loads (Figure S5, Supporting Information), confirming that dehydration occurs even in an aqueous environment. The volume was reduced 5 to 10‐fold, suggesting a limited flow exchange between fibrin gels, extra‐water. The dehydration also densified the fibrin network, leaving more aggregated fibers. The viscoelastic dissipation resulting from those aggregations could contribute to stress concentrations, facilitating crack propagation.

## 4 Experimental Section

### Preparation of Fibrin Hydrogels

High‐concentration fibrinogen was prepared by incubating the fibrinogen powder in DI water at 37°C for 2 h. Fibrin hydrogels were then prepared by mixing fibrinogen (100 mg mL^−1^, Sigma Aldrich), calcium chloride (20 mM, Sigma Aldrich),, thrombin (1 unit for each 500 mg fibrinogen, Sigma Aldrich) in a solution. This mixture was cast into molds created using glass slides before clotting. The molds were subsequently transferred to an incubator set at 37°C for a 30‐min.

### Mechanical Test

Mechanical tests were conducted using a CellScale UniVert equipped with a 10 N load sensor, a water bath. Pure shear tests were employed to determine the fracture energy (Figure S1A, Supporting Information), fatigue threshold (Figure S1B, Supporting Information). For the measurement of fracture energy, each experimental group included two identical samples (width: 25 mm, length between grips: 5 mm, thickness: ≈0.8 mm). These samples were subjected to uniaxial tension at strain rates of 1, 0.5, and 2 s^−1^ until fracture occurred. To identify the critical strain, notched samples with an 8 mm pre‐existing crack were utilized. Unnotched samples were used to obtain the stress‐strain curve. The fracture energy was calculated as the area under the stress‐strain curves up to the critical strain, multiplied by 10 mm. Fatigue threshold measurements involved cyclic loading of samples in each group to the same strain level, at strain rates of 1, 0.5, and 2 s^−1^ for a total of 30 000 cycles. Notched samples were used to monitor crack growth per loading cycle *dc/dN*, while unnotched samples were used to measure the stress‐strain response. The energy release rate *G* was determined as the area under the stress‐strain curves multiplied by 5 mm. The fatigue threshold was defined as the maximum *G* value when no crack growth *dc/dN* = 0 was observed. Uniaxial tensile tests were conducted to determine the ultimate stretch. Dog‐bone shape samples were prepared with a width of 10 mm, a length between two grippers of 20 mm, and, thickness of 1 mm, samples were loaded at a strain rate of 2% /s until fracture.

### Confocal Immunofluorescence

To visualize the fiber morphology, Alexa Fluor 488‐labeled fibrinogen (Thermo Fisher Scientific, USA) was mixed with unlabeled fibrinogen and then clotted. For monotonic fracture analysis, a custom‐made stretching device was used to extend the samples to a stretch ratio of 2.1, fixed in 2% glutaraldehyde for 30 min while still under tension to preserve their structure. For cyclic fatigue‐loaded samples, an initial stretch ratio of 1.5 was applied using a fatigue tester in a dark environment for 1000 cycles. Following this, the samples were transferred to the custom‐made stretching device, stretched to a ratio of 1.5 for fixation. After fixation, the samples were excised from the stretching device, and the notched region was imaged using a Zeiss LSM 710 confocal microscope with a 10× objective lens. The 2D image stacks were captured, subsequently reconstructed into 3D projected images using ImageJ software.

### Attenuated Total Reflectance Fourier Transform Infrared Spectroscopy (AR‐FTIR)

The secondary structures were analyzed using attenuated total reflectance Fourier transform infrared spectroscopy (AR‐FTIR) with a Bruker Vertex V80v Vacuum system equipped with a single‐reflection diamond crystal accessory (Bruker Optik GmbH, Ettlingen, Germany). Spectra were acquired at a scanner velocity of 40 kHz, a resolution of 4.0 cm⁻¹, covering a wavenumber range from 3900 to 400 cm⁻¹. For detailed analysis, the amide I band region (1720–1580 cm⁻¹), which was particularly sensitive to secondary structure, was deconvoluted. This process involved applying a second derivative treatment using a 9‐point smoothing Savitzky‐Golay algorithm. The relative areas under the deconvoluted peaks were then calculated to estimate the proportions of different secondary structural components within the samples.

### Finite Element Analysis (FEA)

FEA simulations were conducted using the open‐source software FEBio. For the unnotched samples, a rectangular strip comprised of hex20 elements was used, with dimensions set at 25 × 5 × 0.8 mm. The notches introduced had dimensions of 8 × 0.5 × 0.8 mm. The upper, lower surfaces of strips were interfaced with rigid bodies. Specifically, the bottom surface was kept fixed, while a prescribed displacement was applied to the top surface in the direction of uniaxial tensile loading. To model monotonic tensile loading, capture fiber alignment prior to fracture, the transversely isotropic Mooney‐Rivlin material model was employed. The uncoupled strain energy density function can be expressed as:

(7) $$\Psi =F_{1}\left(\tilde{I_{1}},\tilde{I_{2}}\right)+F_{2}\left(\tilde{\lambda }\right)+\frac{K}{2}{\left[\ln\left(J\right)\right]}^{2}$$

where fibers exhibited a preferred orientation aligned with the loading direction. The material parameters were obtained by fitting the unnotched model to the stress‐stretch curves from monotonic tensile tests on unnotched specimens. Subsequently, these parameters were used to simulate the behavior of notched samples under uniaxial tensile loading up to a stretch *λ* = 1.4.

For the simulation of cyclic fatigue loading at the 30 000th cycle, a neo‐Hookean hyperelastic material model was selected. Its strain‐energy function is given by:

(8) $$W\;=\frac{\mu }{2}\left(I_{1}-3\right)-\mu \ln\left(J\right)+\frac{\lambda }{2}{\left[\ln\left(J\right)\right]}^{2}$$

Similar to the approach taken for monotonic loading, the unnotched model was first adjusted to match the stress‐stretch data from fatigue tests after 30 000 cycles. The resulting material parameters were then applied to model the response of notched samples under a uniaxial tensile loading to the same stretch *λ* = 1.4.

## Conflict of Interest

The authors declare no conflict of interest.

## Supporting information



Supporting Information

## Data Availability

Data available on request from the authors.
