# Supplementary material for: Molecular Creep Induced Fatigue Rupture of Fibrin Clots
Source: Adv Sci (Weinh). 2025 Aug 7;12(38):e05109. doi: 10.1002/advs.202505109 (PMC12520524; doi:10.1002/advs.202505109)
Supplement: Supplementary file 1 — Supporting Information [file ADVS-12-e05109-s001.docx]

Supporting Information

Molecular creep induced fatigue rupture of fibrin clots

Dani Liu, Zichang Jia, Binchao Liu, Jizhe Hou, Rui Bao,* Mingkun Wang*


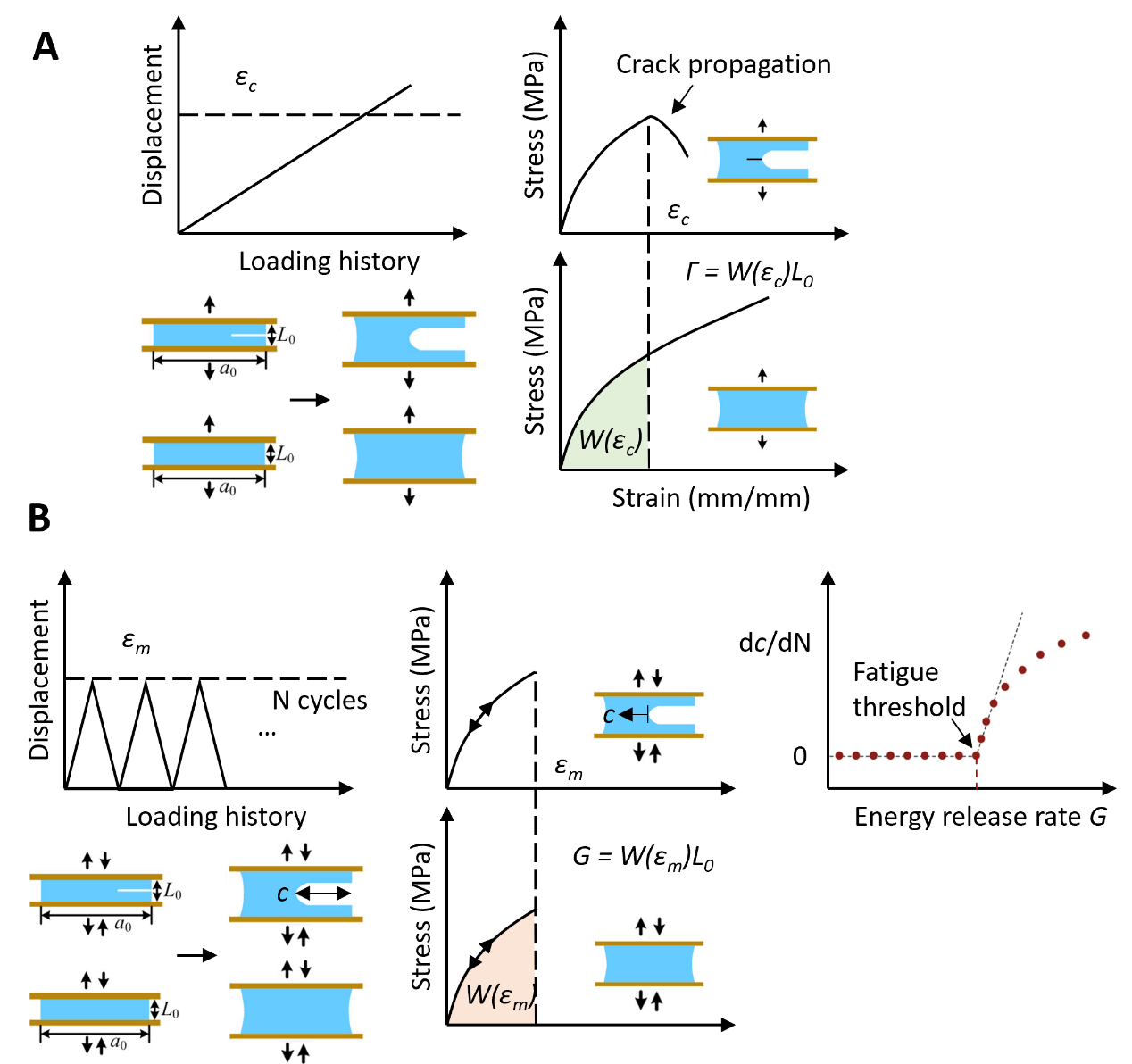


**Figure S1 Pure shear tests for measurements of** **A**. fracture toughness and **B**. fatigue threshold


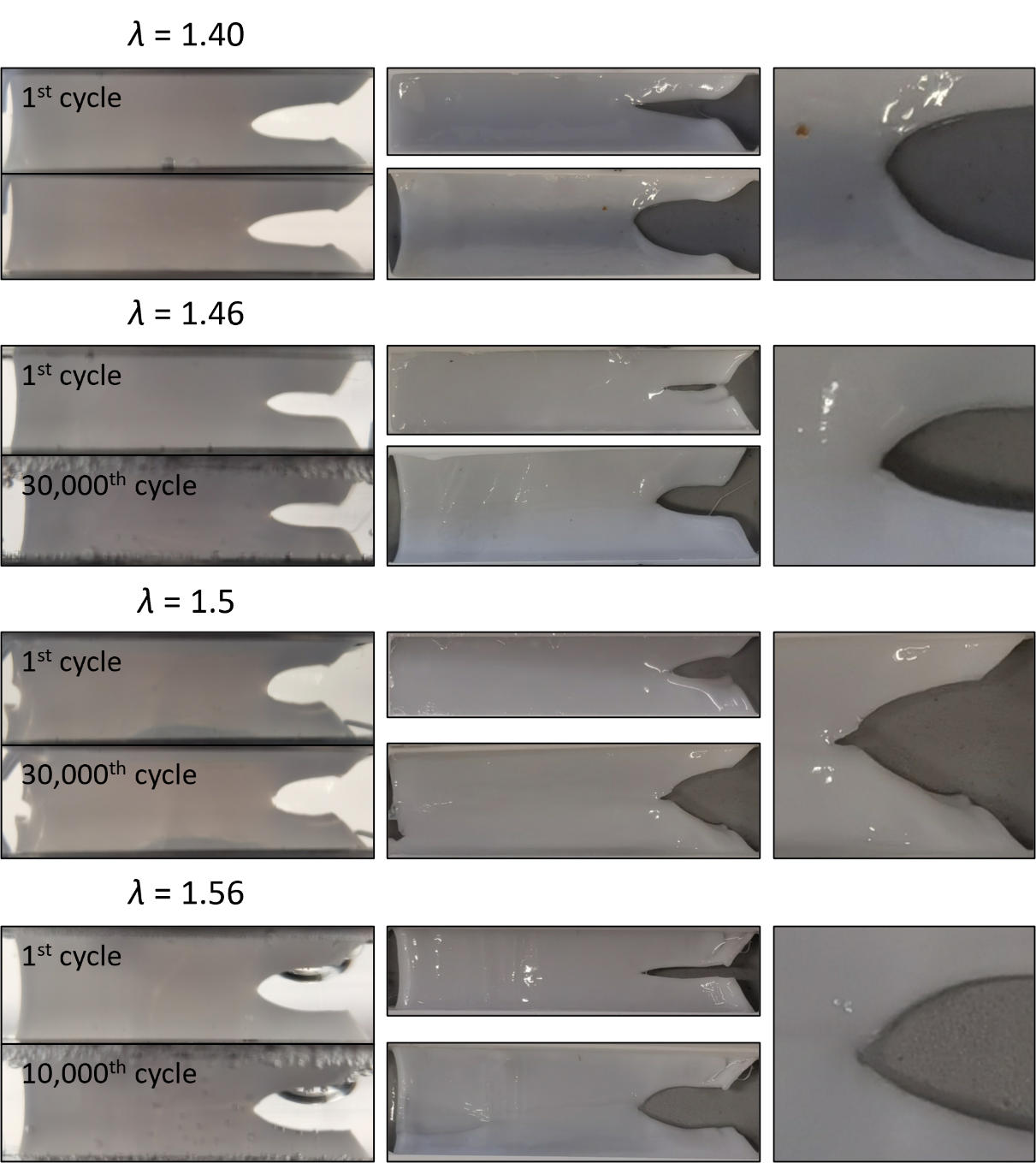


**Figure S2** Photos showing crack growth under cyclic loading to different stretches.


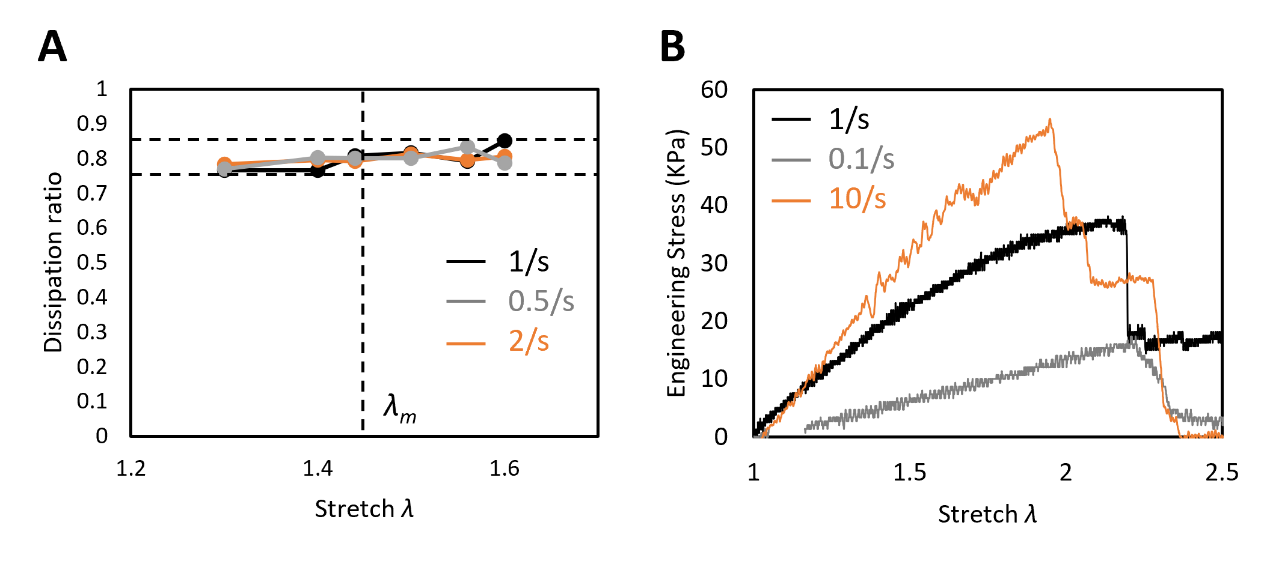


**Figure S3 A**. Ratios of energy dissipation to total strain energy, ranging from *λ* = 1.3 to 1.6, at strain rates of 1/s, 0.5/s, 2/s. **B**. Engineering stress-stretch curves of notched samples under monotonic tensile loading at different strain rates.


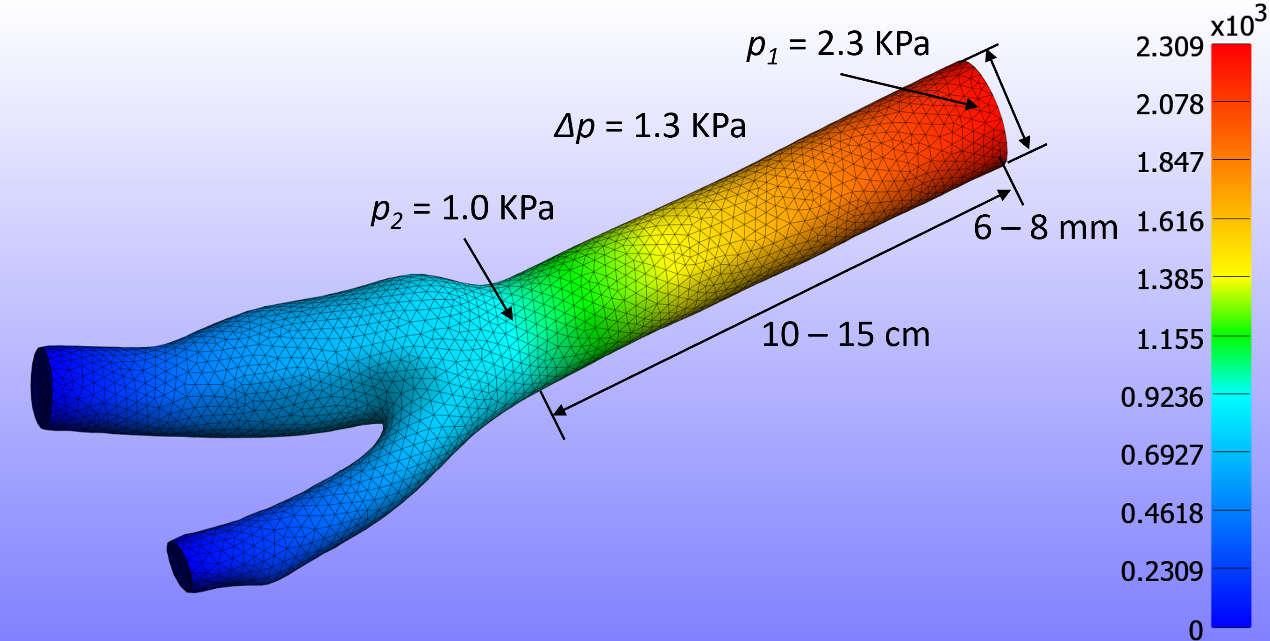


**Figure S4** CFD Modeling of a common carotid artery.


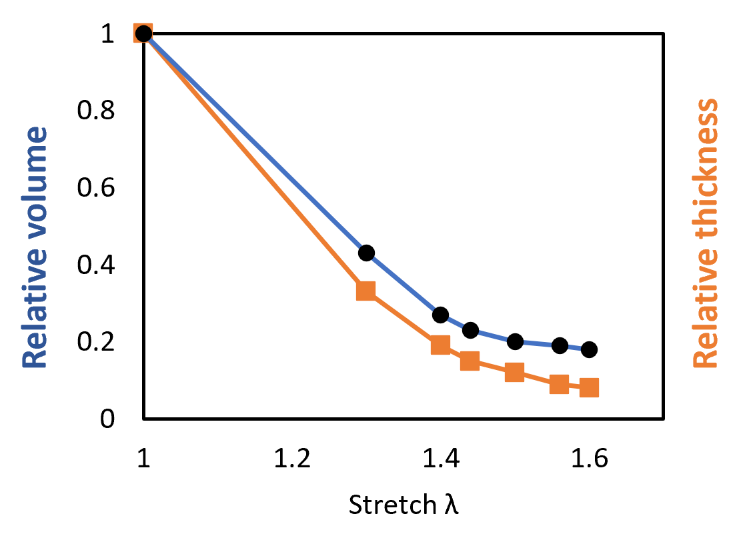


**Figure S5** Relative thickness and volume decrease with increasing stretch under repeated cycles of loading and unloading.
